# For Group II Introns, More Heat Means More Mobility

**DOI:** 10.1371/journal.pbio.1000392

**Published:** 2010-06-08

**Authors:** Richard Robinson

**Affiliations:** Freelance Science Writer, Sherborn, Massachusetts, United States of America

The so-called group II introns perform some neat tricks. They are primarily found within genes and are transcribed into precursor RNA when the host gene is activated. That's when the fun starts. First, a portion of the intron RNA binds a ribosome and dictates production of an “intron-encoded protein,” or IEP. Next the IEP binds to the intron RNA, coaxing it to fold up into a catalytically active structure, a “ribozyme,” which then cuts the intron—that is, itself—out of the precursor RNA. Still bound to the IEP, the intron then seeks out a new home elsewhere in the DNA, base-pairing its sequence to an appropriate target site and splicing itself into one strand of the double-helix. Next, the IEP reverse-transcribes the intron to make the complementary DNA sequence for the other strand. Host enzymes complete the process and tidy up the loose ends, and presto—the intron has copied itself into the genome.

Group II introns are found in bacteria and eukaryotic organelles. They also resemble eukaryotic spliceosomes, molecular machines that remove introns from pre-messenger RNA within the eukaryotic nucleus, and biologists have hypothesized that spliceosomes evolved from group II introns that came aboard with the bacterial ancestors of our mitochondria. But most bacteria carry very few group II introns, suggesting their proliferation is tightly controlled, and it has been unclear how they might have multiplied sufficiently to provide the numbers found in eukaryotes. In this issue of *PLoS Biology*, Georg Mohr, Eman Ghanem, and Alan Lambowitz suggest an answer, one that relies in part on higher temperatures thought to exist at the time that eukaryotes first emerged.

The authors began with the observation that at least one bacterial species, the heat-loving *Thermosynechococcus elongatus*, contains plenty of group II introns—28 in all—amounting to more than one percent of its genome. The 28 are all closely related and constitutes 6 families. Each family has a slightly different target specificity, which is likely one mechanism explaining their ability to proliferate in the bacterial genome.

Three of the 28 introns are only fragments, having lost large chunks of their sequence, but 25 are intact. Of these, eight appear to have complete IEP sequences, while the IEP sequences in 17 have become corrupted and can no longer code for a functional protein. But that doesn't appear to have prevented their proliferation—all 17 of them seem to have descended from a single intron after a deletion corrupted its IEP sequence. That deletion may have only improved their reproductive ability—because they are smaller, they may evade the host cell's defenses more readily. They still need an IEP though, and IEPs from other introns appear to pitch in, having evolved to become less stringent in their specificity for their intron or origin.

The high temperatures preferred by the bacterium don't impair the intron's ability to move within the genome—on the contrary, the mobility efficiency of one intron increased dramatically when the temperature rose from 37°C to 48°C. One likely factor increasing that efficiency is that high temperature increases DNA “melting,” or dissociation of the two DNA strands. This both improves the intron's access to the DNA for base pairing and reduces the role of the IEP in selecting the site, hence increasing the number of potential insertion sites.

The combination of variation in target specificity, reduced size, less stringent IEP specificity, and high temperature appears to account for the greater proliferation of introns within *T. elongatus*.

The authors speculate that this facilitation of intron mobility at high temperatures may echo the situation in which eukaryotes first evolved, believed to be at temperatures between 50°C and 65°C, and may have contributed to the large number of group II descendants found throughout the eukaryotic genome. Over time, early eukaryotic group II introns may have evolved to lose most of their IEPs, leading to the adoption of a common splicing mechanism, which ultimately evolved into the eukaryotic spliceosome.

**Mohr G, Ghanem E, Lambowitz AM (2010) Mechanisms Used for Genomic Proliferation by Thermophilic Group II Introns. doi:10.1371/journal.pbio.1000391**

